# Production of Plant-Based, Film-Type Scaffolds Using Alginate and Corn Starch for the Culture of Bovine Myoblasts

**DOI:** 10.3390/foods13091358

**Published:** 2024-04-28

**Authors:** Jun-Yeong Lee, Jihad Kamel, Chandra-Jit Yadav, Usha Yadav, Sadia Afrin, Yu-Mi Son, So-Yeon Won, Sung-Soo Han, Kyung-Mee Park

**Affiliations:** 1 College of Veterinary Medicine, Chungbuk National University, Cheongju 28644, Republic of Korea; dkujunyeong@naver.com (J.-Y.L.);; 2 School of Chemical Engineering, Yeungnam University, 280 Daehak-ro, Gyeongsan 38541, Republic of Korea; 3 Institute of Cell Culture, Yeungnam University, 280 Daehak-ro, Gyeongsan 38541, Republic of Korea

**Keywords:** alginate, bovine myoblasts, cultured meat, film scaffold

## Abstract

Natural scaffolds have been the cornerstone of tissue engineering for decades, providing ideal environments for cell growth within extracellular matrices. Previous studies have favored animal-derived materials, including collagen, gelatin, and laminin, owing to their superior effects in promoting cell attachment, proliferation, and differentiation compared to non-animal scaffolds, and used immortalized cell lines. However, for cultured meat production, non-animal-derived scaffolds with edible cells are preferred. Our study represents the first research to describe plant-derived, film-type scaffolds to overcome limitations associated with previously reported thick, gel-type scaffolds completely devoid of animal-derived materials. This approach has been employed to address the difficulties of fostering bovine muscle cell survival, migration, and differentiation in three-dimensional co-cultures. Primary bovine myoblasts from *Bos Taurus Coreanae* were harvested and seeded on alginate (Algi) or corn-derived alginate (AlgiC) scaffolds. Scaffold functionalities, including biocompatibility and the promotion of cell proliferation and differentiation, were evaluated using cell viability assays, immunofluorescence staining, and reverse transcription-quantitative polymerase chain reaction. Our results reveal a statistically significant 71.7% decrease in production time using film-type scaffolds relative to that for gel-type scaffolds, which can be maintained for up to 7 days. Film-type scaffolds enhanced initial cell attachment owing to their flatness and thinness relative to gel-type scaffolds. Algi and AlgiC film-type scaffolds both demonstrated low cytotoxicity over seven days of cell culture. Our findings indicated that PAX7 expression increased 16.5-fold in alginate scaffolds and 22.8-fold in AlgiC from day 1 to day 3. Moreover, at the differentiation stage on day 7, MHC expression was elevated 41.8-fold (Algi) and 32.7-fold (AlgiC), providing initial confirmation of the differentiation potential of bovine muscle cells. These findings suggest that both Algi and AlgiC film scaffolds are advantageous for cultured meat production.

## 1. Introduction

Global climate change trends, together with increasing meat consumption, have necessitated the exploration of alternative dietary protein sources to supplement traditional animal meat production [1,2]. Animal agriculture is a significant contributor to global environmental pollution and has resulted in extensive land repurposing for raising and feeding livestock [2,3]. Current industrial animal farming practices underlie deforestation, significant loss of biodiversity, and high greenhouse gas emissions [3,4]. These industries have been implicated in zoonotic disease outbreaks. To combat these diseases, animals are often exposed to large amounts of antibiotics, which exacerbates antibiotic resistance [3,5]. Ethical considerations concerning the husbandry and slaughtering of livestock have been brought to the fore. Given the substandard conditions in which livestock are maintained and the distress that they endure during slaughter, it is imperative to confront these issues. Nevertheless, the prevailing high demand for meat complicates resolving these dilemmas within the current framework of livestock management systems [6]. Cultured meat techniques can address important problems including animal welfare, future food security for human health, infection control, and climate change [7]. These arguments support pursuing cultured meat development as a potential solution.

Several pivotal components should be considered when developing cultured meat production methods: biomaterial selection, scaffold topology, and choice of cells [2,8,9]. Biomaterials have been extensively studied for their applications in cell co-cultures. An ideal scaffold facilitates cell attachment, proliferation, and differentiation [10]. Materials under consideration for application in cultured meat production can be classified as synthetic or natural. Synthetic materials, such as polylactic acid, polyethylene glycol, and poly lactic-co-glycolic acid, incorporate modifications to improve cell adhesion [11,12,13]. However, many synthetic materials are not digestible and exhibit slow degradation rates [14]. Nature-derived materials are thus more suitable for cultured meat. More specifically, nature-derived materials are categorized into three groups based on their chemical structure: polysaccharides, lipids, and proteins [8].

Many previous studies have utilized animal-derived materials, including gelatin, laminin, and collagen, together with polysaccharides such as alginate (Algi), to produce scaffolds owing to their ability to promote cell viability, proliferation, and differentiation [15,16,17]. However, the use of these materials subverts a fundamental objective of cultured meat production.

Most previous studies have reported the application of plant-derived materials such as zein, cellulose, and lignin based on alginate [18]. While these materials can strengthen cultured meat’s physical properties by hardening its scaffold, they have limited ability to enhance texture. To replicate a texture similar to that of actual meat, we chose nontoxic, viscous alginate and cornstarch, which can provide a chewy texture.

Algi, a plant biomaterial belonging to the polysaccharide group, is extensively used in the food industry as a thickener and texturizer [19], and its application in tissue regeneration studies is notable, as it is often used either independently or in combination with other materials due to its biocompatibility [20]. Furthermore, the ability of Algi to readily form gels with bivalent cations such as Ca^2+^ and Zn^2+^, with properties that mimic the extracellular matrix (ECM), has been well demonstrated [16,17,21,22]. Corn starch, comprising linear amylose and amylopectin, is a nontoxic, cost-effective, and widely available biomaterial [23,24,25]. In a few studies, starch blended with Algi has served as a structural component that prevented Algi shrinkage [26,27]. The amylose-to-amylopectin ratio significantly influences the dendritic structure and physical properties, thereby affecting water absorption and swelling during scaffold formation [23]. Studies have demonstrated that starch-based scaffolds facilitate cell attachment, cell proliferation, and differentiation of various cell types [13,28,29,30]. Moreover, byproducts of corn, such as zein protein, are nontoxic to cells [31]. Most studies to date have reported the use of zein protein or corn silk but not starch.

Scaffold properties such as composition, surface topography, chemical properties, and mechanical characteristics significantly influence cellular behavior [9]. Previous studies have investigated the critical role of scaffold surface topography in facilitating cell attachment and spreading, particularly within co-culture systems [32]. Diverse scaffold materials and variations in surface topography result in different levels of stiffness, cellular responses, and morphologies [33]. One study reported that scaffolds with substantial thickness, such as gel-type scaffolds, do not yield optimal conditions, particularly for efficient nutrient and oxygen delivery [34]. Consequently, the development of film-type scaffolds has been pursued [33]. These scaffolds offer advantages, particularly in supporting cell adhesion, proliferation, and differentiation [33,35].

The choice of cell source for cultured meat production is crucial. Because these cells must be edible and capable of proliferating, cells derived from livestock muscle tissue or differentiated muscle cells derived from adult or embryonic stem cells may be suitable cell sources [36]. However, recent meat cultivation research has focused predominantly on immortalized cell lines, such as C2C12 mouse muscle cells, while studies involving the co-cultivation of actual livestock-derived stem cells on scaffolds are lacking [36,37,38].

This study was performed to develop a novel protocol for the fabrication of plant-derived film scaffolds devoid of animal sources and evaluate the adhesion, proliferation, and differentiation of bovine myoblasts within a co-culture system.

## 2. Materials and Methods

### 2.1. Algi-Based Scaffold Preparation

The gel-type scaffold synthesis protocol described by Yu et al., with slight modifications, as compared to the film-type protocol was used [7]. In brief, 2% Algi or 1% cornstarch mixed with 2% Algi (AlgiC) were dissolved in distilled water to prepare scaffold solutions at 21–23 °C. In the gel-type protocol, the solution was frozen in a 3.5 × 2.0 × 1.5 cm mold at −20 °C for 24 h. Subsequently, an 8% CaCl_2_ (Sigma Aldrich, Burlington, MA, USA) solution dissolved in 70% ethanol (Duksan General Science, Seoul, Republic of Korea) was added at −20 °C for another 24 h. Following gelation, the scaffold was sterilized using 70% ethanol at room temperature for 24 h. Finally, the scaffold was washed with phosphate-buffered saline (PBS) and dried at room temperature for 24 h before cell seeding.

Two types of film-type scaffold solutions were prepared using Algi and AlgiC, both purchased from Sigma-Aldrich. These solutions, Algi alone and AlgiC, were dissolved in distilled water at 90 °C until homogeneous. Then, 10 mL of each solution was poured into a 10 cm plate mold, and 2% CaCl_2_ was added to ensure full submersion for a 10 min gelation period. Each gelled scaffold was pressed and rolled using a rolling pin to create a thin film. Both types of scaffolds were sterilized using 70% ethanol containing 2% CaCl_2_ overnight, followed by ultraviolet light for 30 min. After the complete removal of ethanol, the scaffolds were washed twice with autoclaved distilled water. They were then dried at 60 °C for 3 h to completely evaporate the ethanol. Dried scaffolds were subsequently immersed in Dulbecco’s Modified Eagle Medium (DMEM) with high glucose (Thermo Fisher, Waltham, MA, USA) supplemented with 20% fetal bovine serum (FBS, Cytiva, Marlborough, MA, USA), 1% antibiotic-antimycotic (WelGene, Gyeongsan, Republic of Korea), 5 ng/mL of fibroblast growth factor (FGF, PeproTech, Cranbury, NJ, USA), and 20 mM of A MAPK homolog-p38 inhibitor (Adezmapimod, SB 203580, Ann Arbor, Cayman, MI, USA) before seeding cells (Figure S1).

### 2.2. Bovine Myoblast Isolation and Cell Culture

This study was approved by the Institutional Animal Care and Use Committee (IACUC) of the Chungbuk National University (Approval No. CBNUR-1575-21-02).

Fresh tissues from a male Hanwoo cow (*Bos Taurus coreanae*) were obtained from a slaughterhouse and transported to the laboratory on ice. To isolate bovine satellite cells, the protocol described by Kaplan et al. was followed, with slight modifications [34]. In brief, bovine tissues were disinfected with a 2% iodine solution for 10 min. Outer surfaces were trimmed to remove fat and connective tissue. Tissue was minced with sterilized scissors and aliquoted into 50 mL tubes containing 10 mL of 0.2% collagenase/dispase (Sigma Aldrich). These tubes were incubated for 60 to 90 min at 37 °C, with pipette trituration performed every 15 min. The solutions were then triturated several times using an 18-gauge needle until the suspensions became smooth. Next, 20 mL of high-glucose DMEM supplemented with 0.2% primocin (InvivoGen, San Diego, CA, USA), 20% FBS, and 5 ng/mL of FGF was added to each solution. The digests were filtered through both 70 and 40 µm strainers (Falcon Life SCIENCE Ltd., London, UK), then centrifuged at 200× *g* for 5 min. Supernatants were removed, and 20 mL of medium was added. Cells were counted using a hemocytometer, and 7.5 × 10^6^ cells were cultured in uncoated T75 tissue-culture dishes for 24 h. Subsequently, the culture medium was transferred to 0.01 mg/mL laminin (Sigma Aldrich)-coated cell culture dishes and left untouched for 72 h in the incubator. After two weeks of culture, primocin in the growth medium was replaced with a growth medium containing 1% antibiotic–antimycotics. Cells were maintained in DMEM supplemented with 20% FBS, 1% antibiotic–antimycotic, 5 ng/mL of FGF, and 20 nM of SB 203580 in laminin-coated dishes. All cells were incubated at 37 °C in 5% CO_2_, with medium changes every 2–3 days. When the myoblasts reached approximately 100% confluence, the proliferation medium was replaced with a differentiation medium containing 1% antibiotic–antimycotic and 2% horse serum (HS) in DMEM.

### 2.3. Cell Seeding on the Scaffold

To seed bovine myoblasts, a SPLInsrt^TM^ Standing device (SPL Life Science, Pocheon, Republic of Korea) with the filter removed was placed on both the Algi and AlgiC scaffolds, and cells were added into the insert. This insert helped to prevent cell spillage from the scaffold. Bovine myoblasts were seeded onto each scaffold at a density of 1 × 10^5^ cells. A proliferation medium was added until each scaffold was completely submerged. Cells were incubated at 37 °C in a 5% CO_2_ environment. After one day of culture, the inserts were removed. The medium was changed on day 3. Before proliferation and differentiation assays, each scaffold was punched with a 12 mm biopsy punch (Acuderm Inc., Fort Lauderdale, FL, USA).

### 2.4. Weight and Swelling Measurement in Algi and AlgiC Scaffolds

The scaffolds were dried in an oven for 3 h to assess weight changes and the degree of swelling. Scaffold dry weights (W_0_) were recorded before immersing them in distilled water at 37 °C. Wet weight (W_d_) was measured daily until day 7. The percentage swelling was calculated as follows:

Swelling (%) = (W_d_ − W_0_)/W_0_ × 100 (1)

### 2.5. Scanning Electron Microscopy (SEM) of Algi and AlgiC Scaffolds

During scaffold fabrication, SEM images were obtained to examine whether any structural changes were caused by pressing, rolling, or drying. The scaffolds were dried for 12 h, then coated with gold via sputter deposition, and surfaces were observed at 30,000× magnification using SEM (Gemini 560, Oberkochen, Germany).

### 2.6. Live/Dead^TM^ Cell Imaging Kit Assay

The viability of bovine myoblasts in the scaffolds was assessed using a Live/Dead^TM^ Cell Imaging Kit (Thermo Fisher Scientific) according to the manufacturer’s protocol. Medium was completely removed; then, 40 µL of fresh medium and 40 µL of kit solution were added to each scaffold, followed by a 30 min incubation at 37 °C. Images were acquired using a fluorescence microscope.

### 2.7. Cell Counting Kit-8 (CCK-8) Assay

Bovine myoblast viability on Algi and AlgiC scaffolds was assessed using a CCK-8 assay kit (Sigma Aldrich), following the manufacturer’s protocol. Cells cultured in two-dimensional (2D) cell culture plates on day 1 served as controls. Cell viability on the scaffolds was estimated on days 1, 3, 5, and 7. CCK-8 solution was added to a volume equivalent to 10% of the media. The assay was conducted for 3 h in an incubator at 37 °C. Absorbance was measured at 450 nm using an ELISA reader (TECAN Ltd., Männedorf, Switzerland).

### 2.8. RNA Isolation and Reverse Transcription-Quantitative Polymerase Chain Reaction (RT-qPCR)

RNA was isolated using a RNeasy Plant Mini Kit (Qiagen, Hilden, Germany). Purified RNA concentrations were determined using a spectrophotometer. cDNA was synthesized using TOPscript^TM^ RT DryMIX (dN6 Plus) (Enzynomics, Daejeon, Republic of Korea). Data were processed using the ∆∆Ct method, and primers for GAPDH, PAX7, Desmin, MyoD, MyoG, and MHC were used to assess cell proliferation and differentiation (Table 1). Cells cultured in 2D on day 1 were used as controls.

### 2.9. Immunofluorescence (IF) Staining

Cell scaffolds were fixed using BIOFIX HD (BIOGNOST, Zagreb, Croatia) for 15 min. After fixation, cells were treated with 0.2% Triton X-100 for 10 min and washed with 1× PBST. Bovine serum albumin BSA (Sigma-Aldrich) was dissolved in 1× PBST and applied to the scaffolds for 30 min. The BSA solution was then removed. Primary antibodies, namely monoclonal anti-myosin antibody produced in mouse and α-desmin antibody (Sigma Aldrich), were diluted 1:100 and applied for 3 h at room temperature. Primary antibodies were removed and the secondary antibody, goat anti-mouse IgG (Invitrogen, Waltham, MA, USA), was diluted at 1:500 and incubated for 30 min in the dark. Finally, DAPI (Sigma Aldrich) was diluted 1:1000 and used for a 5 min treatment to stain the nuclei.

### 2.10. Statistical Analysis

Data were expressed as means ± standard deviation. One-way analysis of variance (ANOVA), followed by Tukey’s test, was performed using GraphPad Prism software (GraphPad Software 10.2.0, MA, USA). Statistical significance was set at *p* < 0.05.

## 3. Results

### 3.1. Production Time and Thickness Comparison of Gel-Type and Film-Type Algi-Based Scaffolds

**Figure 1:**
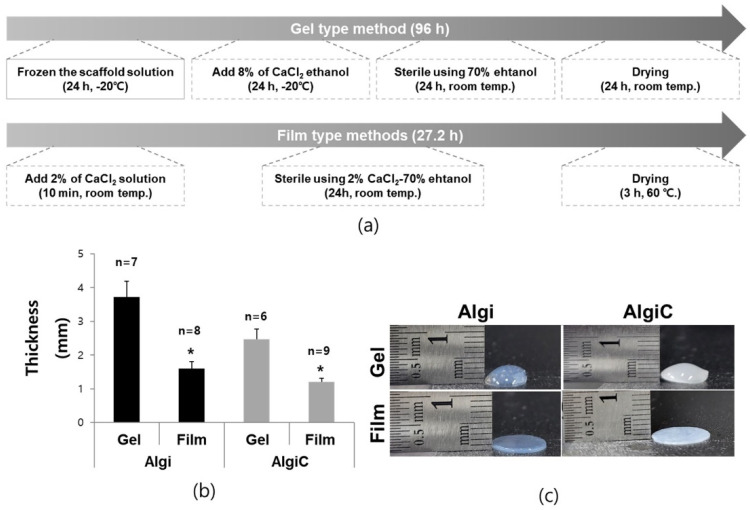
Production time and thickness comparisons of gel-type and film-type Algi-based scaffolds. (**a**) The film-type scaffold preparation time was compared to that of gel-type scaffold preparation. (**b**,**c**) Gel-type scaffolds showed uneven surfaces and lacked flatness, whereas film-type scaffolds exhibited even surfaces and substantially reduced thicknesses (Scale bar = 1 cm, *: *p* < 0.05, *n* = sample number).

**Figure 2:**
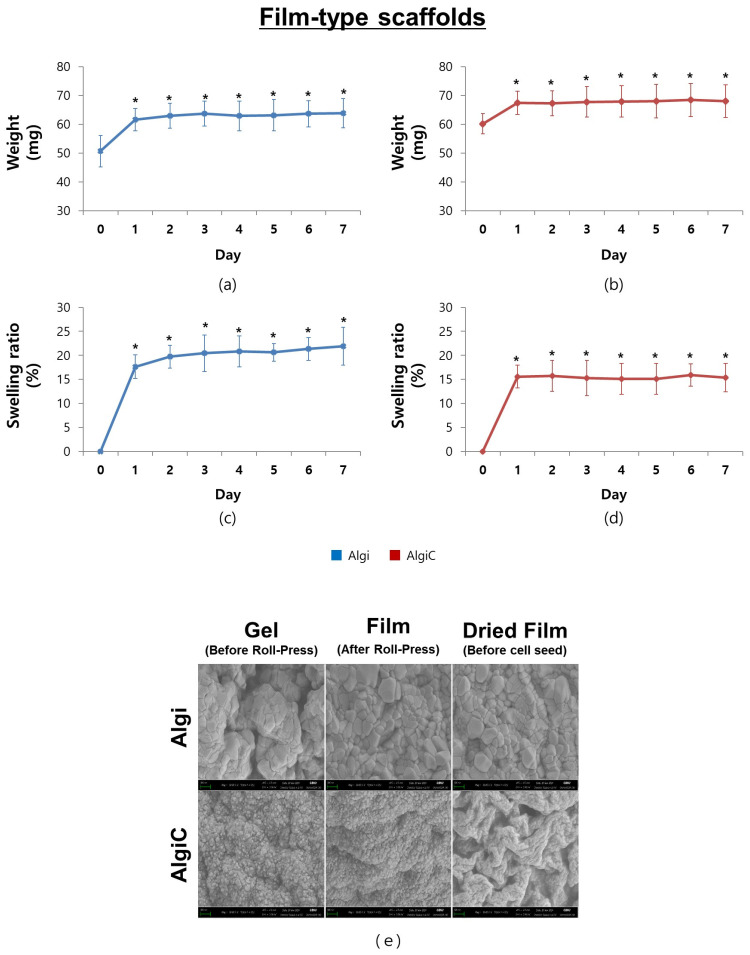
Characterization of alginate (Algi) and alginate–corn starch (AlgiC) scaffolds. (**a**–**d**) Both Algi and AlgiC scaffolds maintained their structures for at least 7 days, as confirmed through weight variation and swelling ratio assays. (**e**) Observation of scaffold surfaces at 30,000× magnification during the manufacturing process (Scale bar = 300 nm, *n* = 16, Algi; *n* = 24, AlgiC; *: *p* < 0.05).

Scaffold production times for the previously reported gel-type scaffold and the film-type scaffold were compared. The reference method required approximately 96 h to prepare a scaffold, whereas the film method took only 27.2 h. Scaffold preparation time was significantly reduced by 71.66% (Figure 1a). The thicknesses and flatnesses of gel- and film-type scaffolds were compared. The respective gel-type scaffold thicknesses were 3.72 ± 0.45 mm and 2.47 ± 0.29 mm for Algi and AlgiC. Both failed to achieve flatness. Film-type scaffolds showed significant respective decreases in thickness, with 1.59 ± 0.20 mm for Algi and 1.21 ± 0.11 mm for AlgiC (Figure 1b,c). We confirmed that no significant surface changes occurred with Algi or AlgiC scaffolds during the press-and-roll or drying processes (Figure 2e).

### 3.2. Characterization of Film Method Results

In weight variation and swelling ratio assays, the weights of both Algi and AlgiC scaffolds reached the stationary phase after one day of immersion in distilled water. The average weights at this phase were 63.07 ± 0.74 mg for Algi and 67.81 ± 0.74 mg for AlgiC scaffolds (Figure 2a,b). The respective average swelling ratios were 20.37 ± 1.37% for Algi and 15.43 ± 0.31% for AlgiC scaffolds (Figure 2c,d). These results indicate that both scaffolds maintained their structures for at least seven days. SEM images confirmed that no structural changes occurred during the scaffold manufacturing process (Figure 2e). In summary, both the Algi and AlgiC scaffolds maintained their structures without significant degradation.

### 3.3. Cell Attachment and Viability Assay

**Figure 3:**
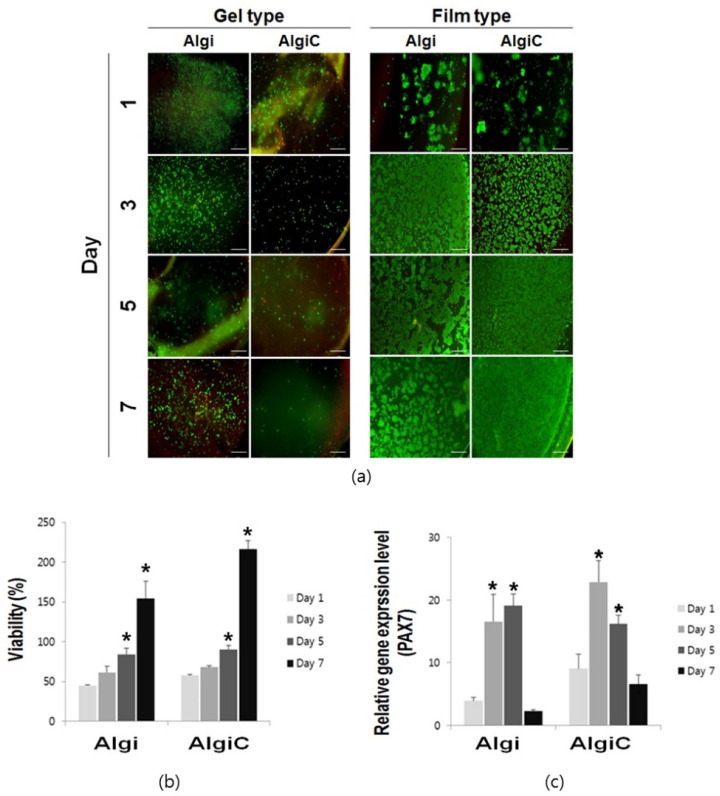
Bovine myoblast attachment and proliferation assays on Algi and AlgiC film-type scaffolds. (**a**) Cell distribution and viability on gel-type and film-type scaffolds were assessed and compared using live/dead staining. (**b**) Bovine myoblasts proliferated in both alginate (Algi) and alginate–corn starch (AlgiC) scaffolds, as assessed using a cell counting kit-8 (CCK-8) assay. (**c**) The proliferation-associated gene Paired box 7 (PAX7) was highly expressed from day 3 to 5 (*n* = 12, Algi and AlgiC; scale bar = 100 μm, *: *p* < 0.05).

We evaluated bovine myoblast attachment, distribution, and proliferation on the scaffolds. Gel-type scaffolds exhibited uneven cell seeding and insufficient penetration, indicating limitations in seeding adequacy and proliferation potential. Conversely, film-type scaffolds exhibited uniform and effective cell seeding, attachment, and proliferation. Both scaffolds reached confluence levels of 84.14 ± 7.83% and 90.10 ± 5.09%, respectively, on day 5, as indicated by live/dead staining and the CCK-8 assay (Figure 3a,b).

Moreover, the CCK-8 assay results indicated that cell viability reached 61.38 ± 7.73% and 84.13 ± 7.83% on the Algi scaffold and 67.81 ± 2.03% and 90.10 ± 5.09% on the AlgiC scaffold on days 3 and 5, respectively. Both Algi and AlgiC scaffolds exceeded confluent cell density by day 7 compared to 2D cultured cells on day 1, used as a control (Figure 3b).

PAX7 expression significantly increased on days 3 and 5 in both Algi and AlgiC scaffolds but significantly decreased by day 7 in both scaffolds (Figure 3c). These results suggest that proliferation was strongly promoted on days 3–5 but subsequently decreased. Thus, plant-based film scaffolds provide an adequate environment for cell attachment and proliferation, with myoblasts surviving for at least 7 days.

### 3.4. Differentiation of Bovine Myoblasts in Algi and AlgiC Scaffolds

**Figure 4:**
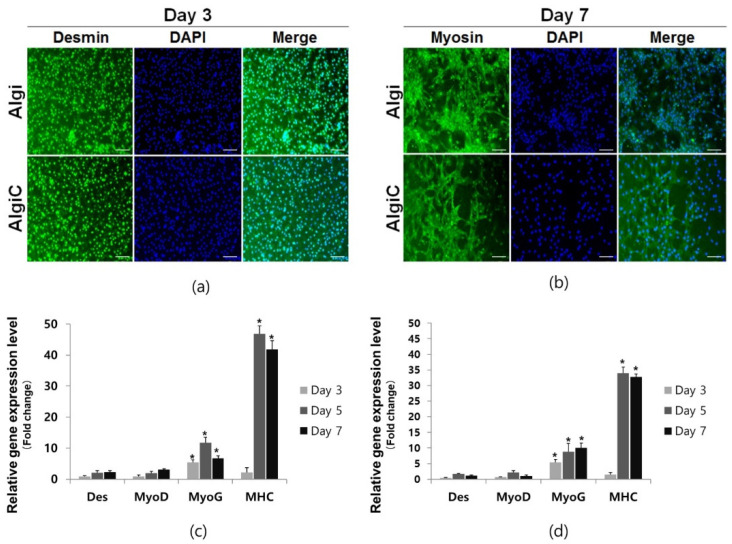
Bovine myoblast differentiation assay on Algi and AlgiC film-type scaffolds. (**a**) Desmin, an early-stage differentiation-related gene, was stained in both Algi and AlgiC scaffolds. (**b**) Myosin, a mature myocyte marker indicating completed differentiation, was expressed in both Algi and AlgiC scaffolds. (**c**,**d**) Differentiation-related genes were expressed, with intermediate and mature stage genes, MyoG and MHC, respectively, showing elevated expression on days 5 and 7 compared to day 1 in a 2D culture (*n* = 20, Algi and AlgiC; scale bar = 200 μm, *: *p* < 0.05).

To evaluate the differentiation capabilities of bovine myoblasts on Algi and AlgiC scaffolds, IF staining and RT-qPCR were performed. For IF staining, myoblasts were stained with a desmin primary antibody upon the initiation of differentiation. During the initial differentiation stage, the myoblasts spread well on the scaffolds (Figure 4a). As myoblast fusion progressed, myofibers formed a mesh-like shape (Figure 4b).

Desmin and MyoD, markers of early-stage differentiation; MyoG, an intermediate-stage marker; and MHC or Myosin, end-stage markers, were analyzed. Relative gene expression results indicated a slight increase in early marker expression, Desmin and MyoD were elevated on both Algi and AlgiC scaffolds on days 5 and 7 compared to day 1. MyoG expression increased on day 5 in both scaffolds. MHC expression significantly increased on both film-type scaffolds on days 5 and 7 (Figure 4c,d). These results suggest that myoblasts can reach final-stage differentiation on film scaffolds.

## 4. Discussion

In this study, we optimized the production of Algi-based film-type scaffolds and confirmed their suitability for bovine muscle cell culture. The plant-based film-type scaffold, composed of Algi with or without corn starch, demonstrated no toxicity and the potential for supporting cell proliferation and differentiation. Previous research has highlighted the use of Algi in tissue engineering because of its biocompatibility, ease of integration with other materials, and structural resemblance to mammal-derived ECM [22,39].

Recent studies have identified three major components essential for cultured meat production: scaffolds, cells, and bioreactors [8]. Various candidate materials for tissue engineering have been identified, including alginate, starch, gelatin, keratin, laminin, polyvinyl alcohol, and polycaprolactone. However, biomaterials should be non-animal-based materials that are digestible by humans and meet the ultimate goal of cultured meat production [10,16,17,21,23,26,28,29,40]. Furthermore, most previous studies utilized mouse cell lines such as C2C12 and L929 or human cell lines such as MG-63 [10,26,29]. Previous studies have focused predominantly on scaffold development, often excluding cell culture integration, and most cellular experiments have been limited to cell lines.

In a study by Xu et al., gelatin-based scaffolds exhibited higher cell viability than scaffolds made from hyaluronic acid, silk fibroin, and chitosan [41]. Scaffolds composed of 10% fish gelatin and 1% sodium alginate treated with Pifithrin and XMU-MP-1 supported robust cell proliferation and the differentiation of piscine satellite cells but not of mammalian cells at 5 × 10^6^ cells/mL.

Purohit et al. investigated a gelatin–Algi–cerium oxide nanocomposite scaffold for bone regeneration using the MG-63 human osteoblast cell line. Gelatin is commonly used in co-culture systems and tissue engineering for its favorable interactions with animal cells in scaffolds containing animal-derived materials [16]. Several studies have been conducted to develop scaffolds using various materials. Goyal et al. described a keratin-containing scaffold, Anna et al. demonstrated a pectin–gelatin scaffold, and Gabriela et al. introduced alginate into a corn starch hydrogel [17,23,40]. However, these studies focused on scaffold physical properties rather than cell experiments.

Lin et al. demonstrated that C2C12, a mouse muscle cell line, could be cultured with different compositions of tapioca starch/Algi scaffolds [10]. At 1 × 10^6^ cells/well, a 1:1 composition resulted in the highest levels of survival, proliferation, and differentiation. However, this study failed to visually confirm cell proliferation and differentiation status.

Myoblasts, known as muscle satellite cells, express PAX7 and Myf5 during their proliferative phase [42,43,44,45]. When proliferation-supporting media were replaced with differentiation media at approximately 100% cell confluence, myoblasts underwent cytoplasmic fusion, forming multinucleated muscle fibers. During this process, genes related to differentiation including MyoD, Desmin, MyoG, and Myosin were expressed (Figure S2) [46,47]. 

This study focused on optimizing the production of Algi-based film-type scaffolds and confirming the potential of bovine myoblasts to sufficiently proliferate and differentiate for applications in cultured meat. Based on previous studies, Algi and corn starch are nontoxic to mammalian cells [10,48]. In our experiments, gel-type scaffolds failed to adequately meet challenges in cell attachment and distribution owing to their uneven surfaces. To overcome this problem, scaffolds were subjected to rolling and pressing to achieve surface uniformity. Moreover, the thin, flat structures of the scaffolds provide advantages for monitoring cell states. The film-type scaffolds exhibited uniform cell distribution, stable attachment, and robust proliferation. Myoblasts showed enhanced proliferation and viability on both Algi and AlgiC scaffolds. Upon starvation and subsequent exposure to a differentiation medium containing 2% HS, the myoblasts underwent differentiation, leading to the formation of multinucleated fibers on the scaffolds. These results indicate the potential of bovine myoblasts to proliferate and differentiate on both Algi and AlgiC scaffolds.

The limitations of this study include the inability to achieve directional differentiation phenotypes compared with those observed in real meat. Culture system scale-up represents a significant challenge. The cell culture media used in this study contained animal-derived materials, including FBS, necessitating the exploration of non-animal-based media alternatives. Potential solutions to overcome these limitations include imprinting parallel patterns on scaffolds and introducing a magnetic field within the medium to direct muscle growth [8,49]. In addition, stacking thin-film scaffolds may be a viable approach for generating thicker tissue structures. Assuming that the scaffolds currently researched are implemented as cultured meat with a diameter of 10 cm and a thickness of 1 cm, using the stacking method, there is a significant low-cost advantage of USD 0.1. However, regulations vary by country, and cultured meat prohibitions exist in a few countries to protect the existing livestock industry, necessitating the relaxation of such laws. Furthermore, it is expected that consumer acceptance can be increased through research using red plant-based materials to achieve a color similar to real meat and through multi-culture studies to implement texture and taste. Further research on texture analysis after scale-up is required.

## 5. Conclusions

In this study, we successfully optimized the production of plant-based film-type scaffolds for bovine myoblast culture, reducing preparation time while maintaining scaffold structure integrity. Myoblast viability, proliferation, and differentiation potential were confirmed up to day 7. Algi and AlgiC scaffolds exhibited swelling ratios of 20.37% and 15.43%, respectively, indicating stability without degradation up to day 7. Our findings demonstrate that primary bovine myoblasts can proliferate and differentiate on animal component-free plant-based scaffolds. Moreover, this co-culture method holds promise for tissue regeneration engineering applications. Additionally, these thin-film scaffolds offer versatility for diverse surface designs or coatings with various substances, facilitating tissue engineering research and regenerative therapy. This study is poised to significantly advance the development of cultured meat.

## Figures and Tables

**Table 1:** Primer nucleotide sequences.

Gene	Direction	Sequence	Tm (°C)	Product Size
GAPDH	Forward	5′-GGTGAAGGTCGGAGTGAACG-3′	60.5	247
	Reverse	5′-GATGTTGGCAGGATCTCGCT-3′		
PAX 7	Forward	5′-GTGCCCTCAGTGAGTTCGAT-3′	59.5	152
	Reverse	5′-TCCAGACGGTTCCCTTTGTC-3′		
Desmin	Forward	5′-CCTCACTGCCTCCTAAAGCC-3′	60.0	192
	Reverse	5′-CAGGCCCCCTCACTTCAAAA-3′		
MyoD	Forward	5′-ATGACCCGTGTTTCGACTCC-3′	60.0	205
	Reverse	5′-TTGCAGGCCCACAGTAAACA-3′		
MyoG	Forward	5′-GCGCAGACTCAAGAAGGTGA-3′	60.0	125
	Reverse	5′-GCAGGCGCTCTATGTACTGG-3′		
MHC	Forward	5′-AAGCTGATGCCTTGGCTGAT-3′	60.0	237
	Reverse	5′-TCTCTGTGGCGTGTTTCTCC-3′		

## Data Availability

The original contributions presented in the study are included in the article/Supplementary Material, further inquiries can be directed to the corresponding author.

## Supplementary Materials

The following supporting information can be downloaded via this link: https://www.mdpi.com/article/10.3390/foods13091358/s1, Figure S1: The scheme of the whole experiment process; Figure S2: Immunofluorescence stain results on day 1 in a 2D culture system.

## Abbreviations

Algi	Alginate scaffold
AlgiC	Alginate–corn starch scaffold
IF	Immunofluorescence
MHC	Myosin heavy chain
MyoD	Myoblast determination protein 1
MyoG	Myogenin
PAX7	Paired Box 7
FGF	Fibroblast growth factor
*n*	Experimental number
