# Supplementary figures and images for: Production of Plant-Based, Film-Type Scaffolds Using Alginate and Corn Starch for the Culture of Bovine Myoblasts
Source: Foods. 2024 Apr 28;13(9):1358. doi: 10.3390/foods13091358 (PMC11083433; doi:10.3390/foods13091358)

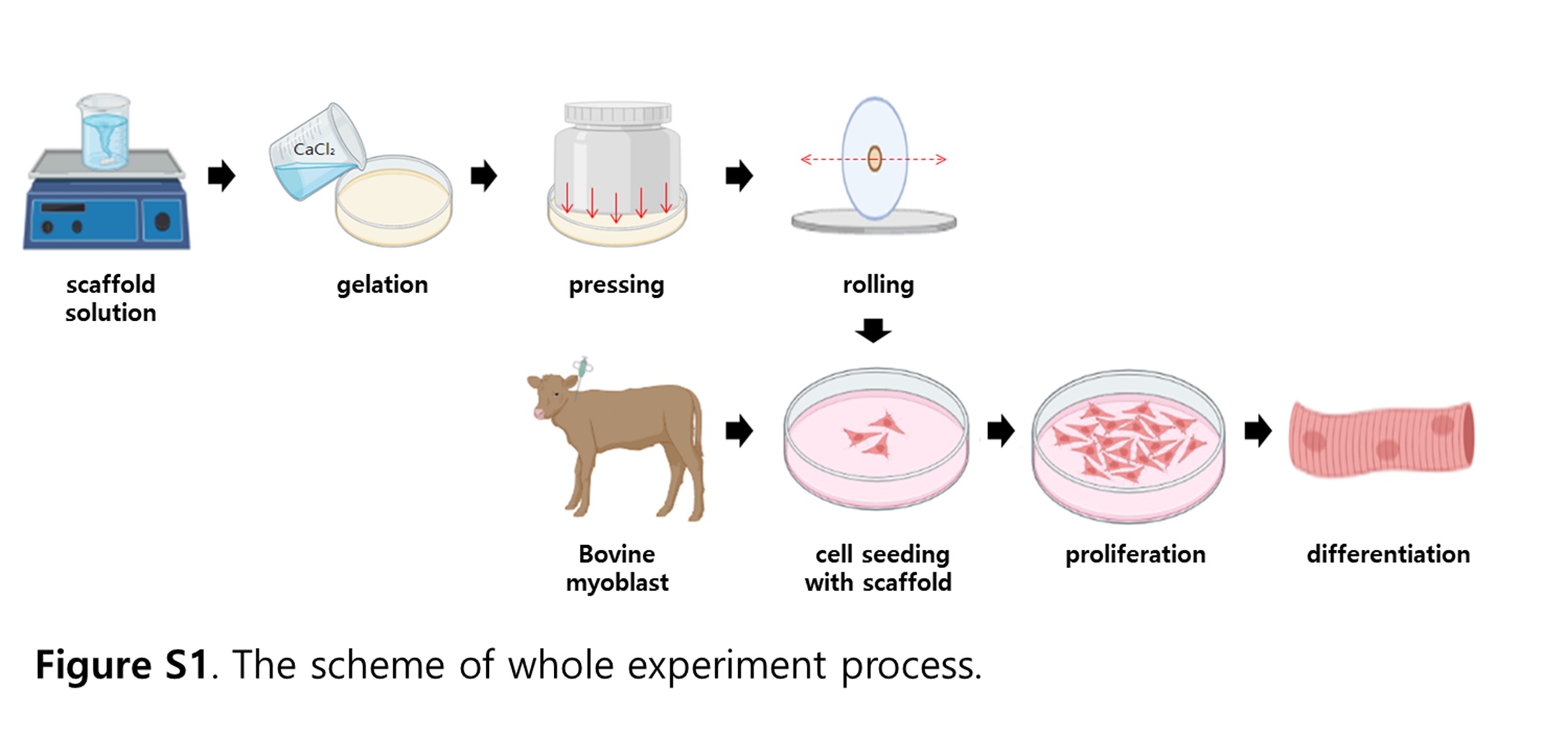

Supplement: Supplementary file 1 [file foods-13-01358-s001.zip › Figure S1.jpg]

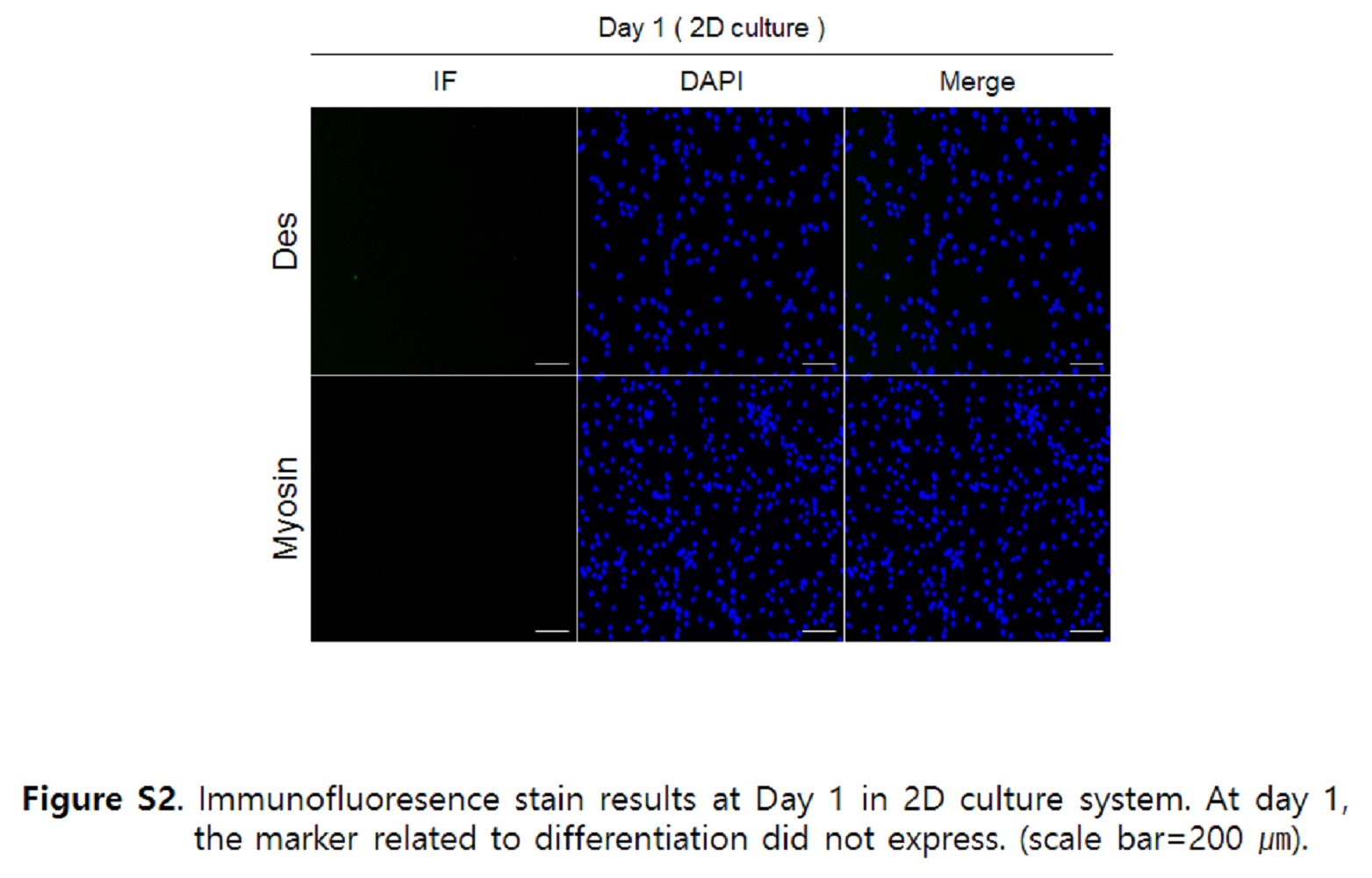

Supplement: Supplementary file 1 [file foods-13-01358-s001.zip › Figure S2.jpg]
